# Novel *CNGA3* and *CNGB3* mutations in two Pakistani families with achromatopsia

**Published:** 2010-04-29

**Authors:** Maleeha Azam, Rob W.J. Collin, Syed Tahir Abbas Shah, Aftab Ali Shah, Muhammad Imran Khan, Alamdar Hussain, Ahmed Sadeque, Tim M. Strom, Alberta A.H.J. Thiadens, Susanne Roosing, Anneke I. den Hollander, Frans P.M. Cremers, Raheel Qamar

**Affiliations:** 1 Department of Biosciences, COMSATS Institute of Information Technology, Islamabad Pakistan; 2 Department of Human Genetics, Radboud University Nijmegen Medical Centre, Nijmegen, The Netherlands; 3 Department of Ophthalmology, Radboud University Nijmegen Medical Centre, Nijmegen, The Netherlands; 4 Nijmegen Centre for Molecular Life Sciences, Radboud University Nijmegen Medical Centre, Nijmegen, The Netherlands; 5 Institute of Human Genetics, Helmholtz Zentrum München, German Research Center for Environmental Health, Neuherberg, Germany; 6 Department of Ophthalmology, Erasmus Medical Centre, Rotterdam, The Netherlands; 7 Shifa College of Medicine, Islamabad, Pakistan

## Abstract

**Purpose:**

To identify the genetic defect in two Pakistani families with autosomal recessive achromatopsia.

**Methods:**

Two families (RP26 and RP44) were originally diagnosed with retinal dystrophy based upon their medical history. To localize the causative genes in these families, homozygosity mapping was performed using Affymetrix 10K single nucleotide polymorphism (SNP) arrays. Sequence analysis was used to find the mutations in candidate genes cyclic nucleotide-gated channel alpha-3 (*CNGA3*; family RP26) and cyclic nucleotide-gated channel beta-3 (*CNGB3*; family RP44). Control individuals were analyzed by allele-specific PCR for the *CNGA3* mutation and BstXI restriction analysis for the *CNGB3* mutation. After genetic analysis, clinical diagnosis was re-evaluated by electroretinography and color vision testing. During the course of this study, selected affected members of family RP26 were given pink glasses as supportive therapy.

**Results:**

Sequence analysis of the positional candidate genes identified a novel missense mutation in *CNGA3* (c.822G>T; p.R274S) in family RP26, and a novel *CNGB3* frameshift mutation (c.1825delG; p.V609WfsX9) in family RP44. Clinical re-evaluation after genetic analysis revealed that both families have segregating autosomal recessive achromatopsia.

**Conclusions:**

Genetic analysis of two Pakistani families with retinal disease enabled the establishment of the correct diagnosis of achromatopsia. Two novel mutations were identified in *CNGA3* and *CNGB3* that are both specifically expressed in cone photoreceptors. Re-evaluation of the clinical status revealed that both families had achromatopsia. The use of pink glasses in patients was helpful in reducing photophobia and enabled rod-mediated vision.

## Introduction

Achromatopsia (ACHM; OMIM 216900) is a congenital autosomal recessive cone disorder with a prevalence of 1 in 30,000 individuals [1]. The clinical features include low visual acuity, nystagmus, photophobia, severe color vision defects, and no recordable or only residual cone function on electroretinography (ERG) with normal rod functions. Fundoscopy is usually normal, although macular pigmentary changes and atrophy have been described in the literature [2-5]. ACHM is characterized by progressive cone loss, which has been described in a large proportion of patients in terms of the worsening of the macular appearance and deterioration of the central vision irrespective of the genetic cause [5,6]. It has been reported that the use of red contact lenses or red tinted glasses can alleviate photophobia in patients with ACHM or cone dystrophy [7,8].

To date, ACHM has been described as being caused by mutations in four genes: cyclic nucleotide-gated channel alpha-3 (*CNGA3),* cyclic nucleotide-gated channel beta-3 *(CNGB3),* guanine nucleotide-binding protein, alpha-transducing activity polypeptide 2 *(GNAT2),* and phosphodiesterase 6C (*PDE6C)* [9-14]. The proteins encoded by these four genes are specifically expressed in cone photoreceptor cells, where they have been shown to be involved in the cone phototransduction cascade. *CNGA3* encodes the α-subunit and *CNGB3* the β-subunit of the cyclic nucleotide-gated (CNG) channel that has a central function in signal transduction of the visual pathway [15]. 25% of all ACHM patients carry mutations in *CNGA3*, and 45%–50% carry *CNGB3* mutations, whereas only a few families have been reported to have *GNAT2* or *PDE6C* mutations [14-18]. Mutations in *CNGA3*, *CNGB3,* and *PDE6C* have also been associated with cone dystrophy in a small proportion of patients [10,14].

In the present study, we report two Pakistani families (RP26 and RP44) which were initially classified as having retinal dystrophy, but after performing genetic analysis they were reclassified as having ACHM.

## Methods

### Initial diagnosis

Two families (RP26 and RP44) were collected from different areas of Pakistan. Family RP26 was collected from the Punjab province, while RP44 was collected from the North West Frontier Province. After documenting the initial clinical histories, both families were initially diagnosed to suffer from retinal dystrophy.

### Ethics declaration

This study conforms to the Helsinki declaration and was approved by the Shifa College of Medicine/Shifa International Hospital Ethics Committee/Institutional Review Board (Islamabad, Pakistan). All patients were informed in their local language of the purpose of the study, and gave their informed written consent before they were further analyzed.

### Genetic analysis

#### DNA isolation and marker analysis

**Figure 1:**
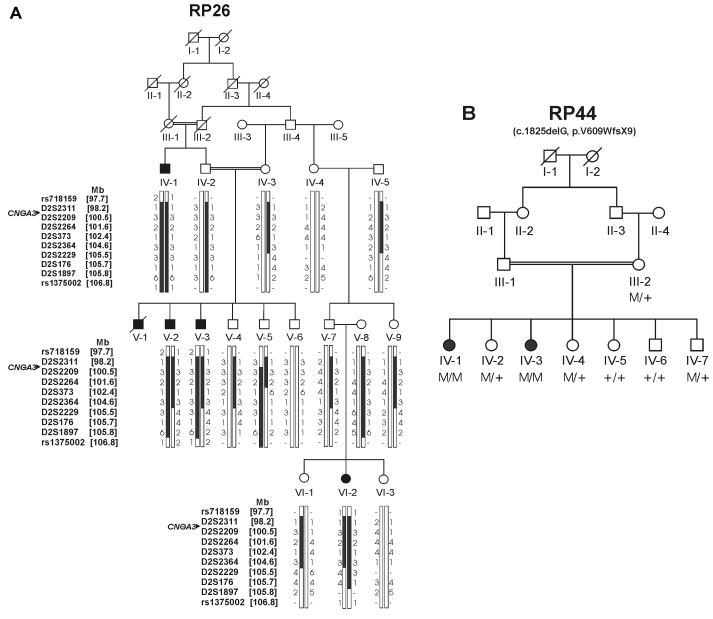
Pedigrees and genotyping results of Pakistani achromatopsia (ACHM) families RP26 and RP44. **A**: Pedigree with marker haplotypes for cyclic nucleotide-gated channel alpha-3 (*CNGA3)* at 2q11.2 for family RP26. White circles represent healthy females, filled circles affected females, white squares healthy males, and filled squares affected males. Deceased individuals are shown with a slanting line across the symbol. Haplotypes were constructed for a 9.1-Mb interval on chromosome 2q11.2, encompassing the *CNGA3* gene (indicated with an arrowhead). **B**: Family RP44 and segregation of the c.1825delG mutation (M) in *CNGB3* (+ represents the wild type alleles).

For family RP26, blood samples were collected from 16 individuals (four affected and 12 healthy individuals) from a six generation pedigree (Figure 1A). For family RP44, blood samples were collected from eight individuals (two affected and six normal individuals) from a four-generation pedigree (Figure 1B). DNA isolation was performed as described previously [19]. All the affected members of family RP26 (IV-1, V-2, V-3, VI-2), and seven individuals of family RP44 (healthy individuals III-2, IV-2, IV-4, IV-6, IV-7 and affected individuals IV-1 and IV-3) were genotyped using the Affymetrix 10K single nucleotide polymorphism (SNP) array containing 10,204 SNPs (Affymetrix, Santa Clara, CA).

Multipoint parametric linkage analysis of the SNP array data of both families was performed using the GeneHunter program in the EasyLinkage software package (version 5.02) [20]. Fine-mapping of the region on chromosome 2q11.2 with the highest logarithm (base 10) of odds (LOD) score in family RP26 was conducted using microsatellite markers that were amplified by the polymerase chain reaction (PCR) under standard conditions. Haplotypes were constructed based upon the size of the alleles of the microsatellites. The positions of the microsatellite markers were derived from the Marshfield map. Two-point parametric LOD scores for the microsatellite markers were calculated using the SuperLink program (version 1.6) in the EasyLinkage software package [20].

### Sequencing of candidate genes

The sequencing primers for the candidate genes *CNGA3* and *CNGB3* have been described before [5]. For family RP26, the seven coding exons of the *CNGA3* gene were sequenced in one affected individual (V-2) along with a control sample, and for family RP44, all 18 coding exons of the *CNGB3* gene were sequenced in individual IV-1 as well as a control sample. The amplified PCR products were separated on agarose gel and purified with a Nucleospin DNA extraction kit (Nucleospin Extract II, Macherey-Nagel GmbH and Co, Germany). Purified products were directly sequenced using the corresponding primers and dye-termination chemistry (BigDye Terminator, version 3 on a 3730 or 2100 DNA analyzer; Applied Biosystems, Foster City, CA).

### Segregation analysis and panel screening

Segregation of the *CNGA3* mutation in family RP26, and analysis of the mutation in 22 unrelated probands with autosomal recessive retinal dystrophies and 150 ethnically-matched control individuals, was performed via allele-specific PCR [21]. For this purpose, three primers were designed; a forward wild type (5′-AAA GGT GGG CAC AAA CTA CCC AGA AGT GAG G-3′), forward mutant (5′-AAA GGT GGG CAC AAA CTA CCC AGA AGT GAG T-3′) and a common reverse primer (5′-AAT GGC AAA GTA GAT GCA GGC ATT CCA GTG G-3′). DNA was amplified using 0.5 mM dNTPs, 1.5 mM MgCl_2_, 0.3 μM of each forward and reverse primer, and 2.5 U Taq polymerase. The thermal cycling conditions were as follows: initial denaturation at 95 °C for 5 min, followed by 35 cycles at 95 °C for 30 s, 60 °C for 30 s, and 72 °C for 30 s, with a final extension cycle at 72 °C for 6 min.

Segregation analysis for the *CNGB3* mutation in family RP44 was performed by direct sequencing of exon 16. Screening of 44 probands with retinal dystrophies for this *CNGB3* mutation was performed by amplification of exon 16 (357 bp) followed by BstXI digestion of the amplified products. Wild-type alleles were digested into 183 bp and 174 bp fragments, whereas mutant alleles remained undigested.

### Clinical re-evaluation

Due to the specific genetic defect that was identified, the affected members of family RP26 were clinically revisited and a detailed clinical examination was performed to confirm the status of the disease in the family. Fundoscopy and electroretinogram (ERG) data were obtained for both families. Visual acuity tests were performed for the affected individuals (IV-1, V-2, and VI-2) of family RP26, and they were also subjected to color differentiation tests using the standard Ishihara plates. Family RP44 could not be revisited for the visual acuity test, but a detailed clinical history was documented from the proband (IV-1) of the family. The affected members of family RP26 were also given pink glasses to use for the alleviation of photophobia.

## Results

### Initial clinical results

Initially, both families (RP26 and RP44) were diagnosed as having retinal dystrophy. This diagnosis was based on their clinical history, because the patients complained about vision loss and nystagmus.

### Homozygosity mapping

In family RP26, whole-genome SNP array analysis revealed a homozygous interval of 9.1 Mb at 2q11.2 between SNP rs718159 and rs1375002 in all the affected members, with a multi-point LOD score of 3.81 at theta 0. Confirmation and refinement of this region was performed by analyzing microsatellite markers between flanking SNPs rs718159 and rs1375002. As a result, the critical region for the underlying defect was refined from the initial 9.1 Mb to 7.2 Mb, flanked by markers rs718159 and D2S2229 (Figure 1A). This region contains the *CNGA3* gene, which has previously been implicated in ACHM and cone dystrophy [9]. In family RP44, homozygosity was observed at nine different chromosomal regions in both affected members (IV-1, IV-3), with a LOD score of 1.22 at theta 0, whereas the unaffected individuals III-2, IV-2, IV-4, IV-6, and IV-7 were heterozygous in these regions. The *CNGB3* gene, which has also previously been shown to be involved in ACHM and cone dystrophy, resides in one of these regions on chromosome 8q21.3 [11].

### Sequence analysis

**Figure 2:**
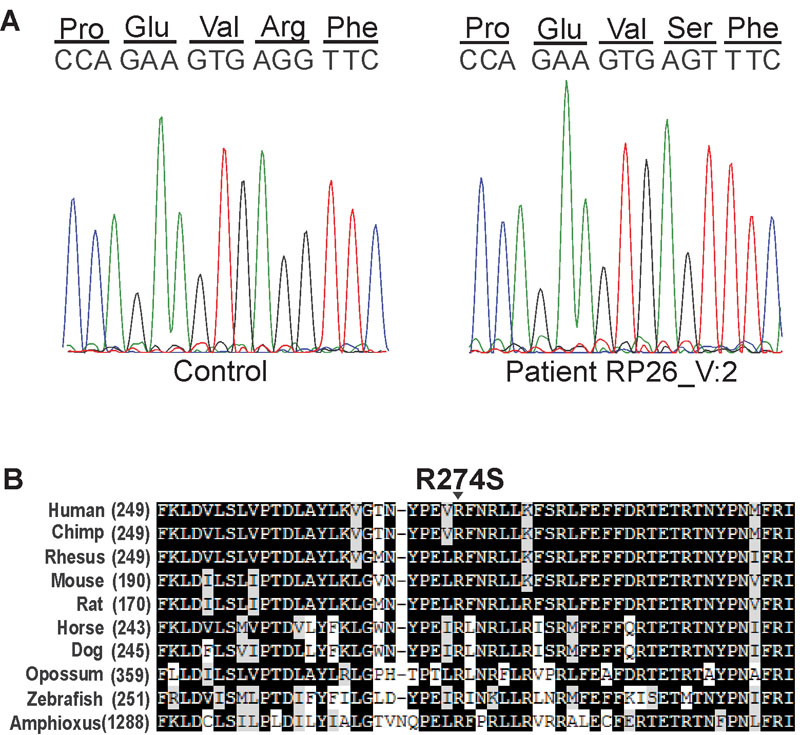
Sequence analysis and amino acid conservation of the mutated cyclic nucleotide-gated channel alpha-3 (CNGA3) amino acid residue in family RP26. **A**: Sequence trace of part of exon 8 in a normal control individual (left panel) and an affected individual (V-2; right panel) showing the homozygous mutant sequence c.822G>T (p.R274S). **B**: Protein sequence alignment of CNGA3 in different species. The mutated arginine residue is highly conserved. The conserved amino acids between different species are shown in black boxes, and the less conserved amino acids are highlighted in light gray or white boxes.

Sequence analysis of all the coding exons of the *CNGA3* gene in the proband of family RP26 revealed a novel change of nucleotide G>T at position 822 (c.822G>T) in exon 8 in the affected family member V-2. This nucleotide change results in the substitution of a serine residue for an arginine residue (p.R274S; Figure 2A). The mutation segregates with the disease in the family, and the mutation was not detected in 22 unrelated probands with autosomal recessive retinal dystrophies, or in 150 ethnically-matched controls.

**Figure 3:**
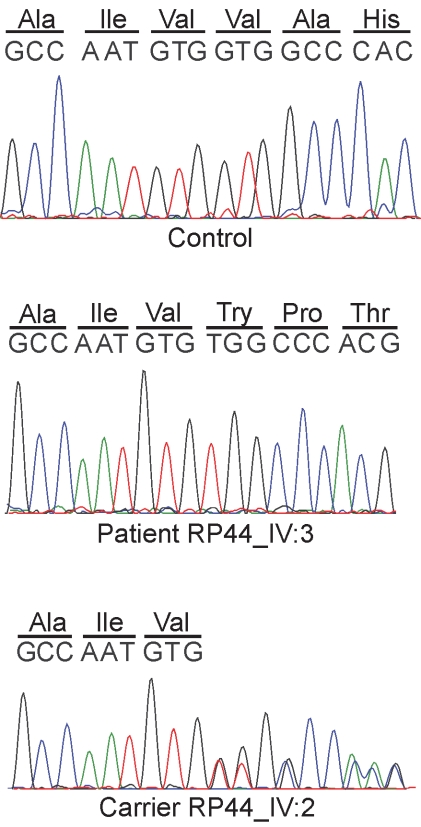
Sequence analysis of cyclic nucleotide-gated channel beta-3 (*CNGB3)* in family RP44. Sequence trace of part of exon 16 in a normal control individual (upper panel), an affected individual carrying a homozygous c.1825delG mutation (middle panel), and a heterozygous carrier of the mutation (lower panel).

Sequencing of all the coding exons of the *CNGB3* gene in the proband of family RP44 resulted in the identification of a novel mutation, a one nucleotide deletion at position 1825 in exon 16 of the gene c.1825delG; Figure 3A), which results in a frameshift and premature termination of the protein (p.V609WfsX9). Segregation analysis in the family revealed that the two affected individuals, IV-1 and IV-3, are homozygous for the mutation, while four healthy persons (III-2, IV-2, IV-4, IV-7) are heterozygous carriers, and two healthy persons (IV-5, IV-6) are homozygous for the wild type allele (Figure 1B and Figure 3A). The c.1825delG mutation was not identified in 44 probands with retinal dystrophies.

### Clinical re-evaluation

**Figure 4:**
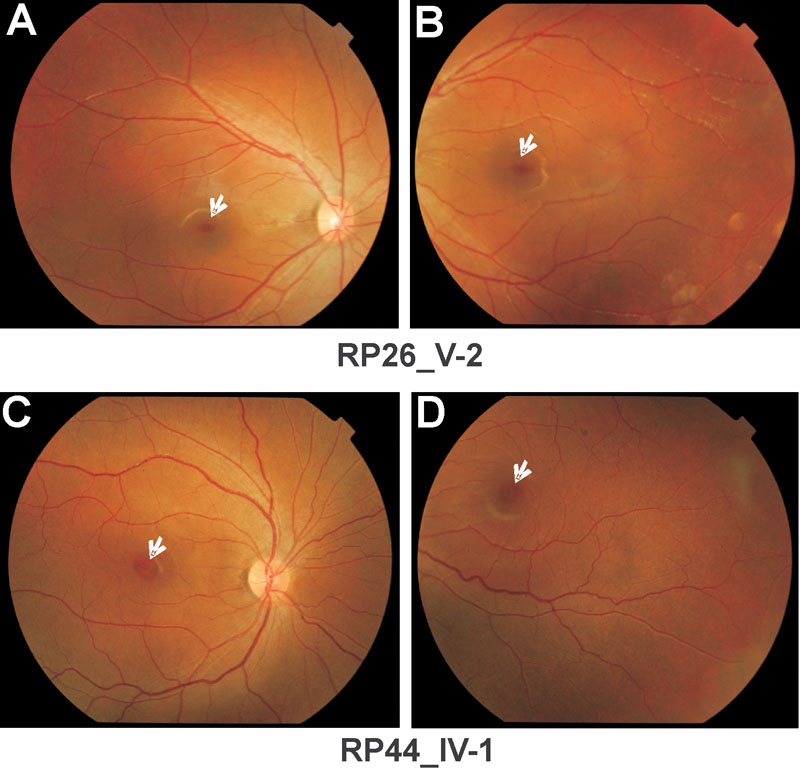
Fundus photographs of affected individuals from families RP26 and RP44. Fundus photographs of affected individual V-2 of family RP26 (**A, B**) and IV-1 of family RP44 (**C, D).** Patient RP26_V-2 and patient RP44_IV-1 show the beginning signs of a Bull’s eye maculopathy (indicated with an arrowhead).

**Figure 5:**
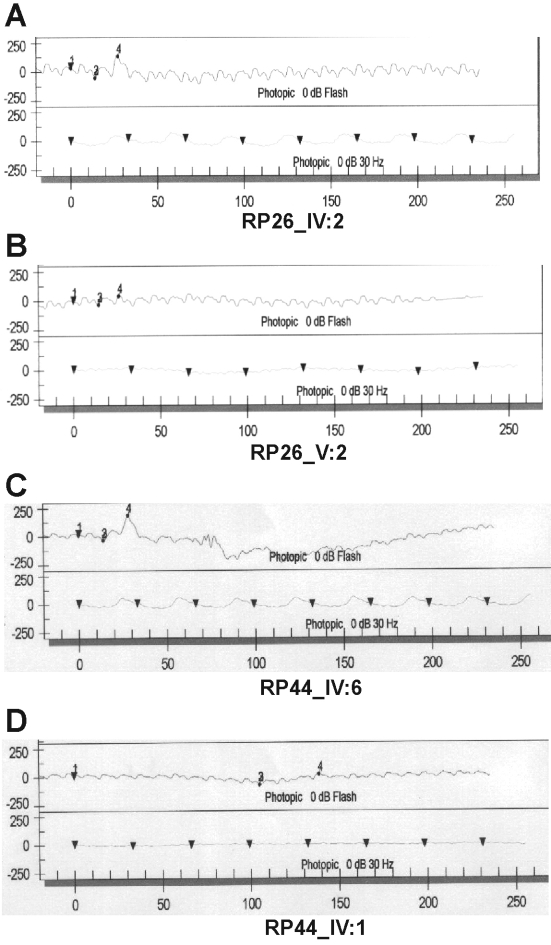
Electroretinogram (ERG) of the normal and affected members of the families RP26 and RP44. The photopic ERGs (**A**, **C**) of the unaffected individuals (RP26_IV:2, RP44_IV:6) of families RP26 and RP44 indicated normal cone responses. In comparison, the affected individuals (RP26_V:2, RP44_IV:1) of both families had residual cone responses (**B**, **D**).

As it has previously been reported that mutations in *CNGA3* and *CNGB3* are associated with cone dysfunction [9-11], the clinical status of the affected individuals from both families was re-evaluated. The affected individuals of family RP26 (IV-1 and VI-2), who were aged 35 and 11 years, respectively, at the time of fundoscopy, showed a normal macular region. However, one affected individual from each family (RP26_V-2 [age 14 years] and RP44_IV-1 [age 19 years]) showed the beginning of a Bull’s eye maculopathy (Figure 4), and revealed some residual cone responses on the photopic ERG on the 30 Hz flicker stimulation (Figure 5), showing that the disease is progressive in these patients.

The affected individuals from family RP26 had congenital nystagmus, photophobia and were color blind. In addition, the affected individuals (IV-1, V-2, and VI-2) had low visual acuity of 20/200, 20/400, and 20/200, respectively. Members of family RP44 could not be revisited for the visual acuity test because they live in a very remote rural area. In this family, a detailed telephone interview with both affected members IV-1 and IV-3 revealed that they also had congenital nystagmus. In addition, they had photophobia along with low visual acuity and were found to be color blind, thereby confirming the molecular diagnosis of ACHM.

In previous studies, affected individuals with cone-dysfunction have been treated with red colored-tinted glasses or red contact lenses to reduce photophobia [7,8]. To determine whether this would also help in family RP26, the affected individuals (IV-1, V-2) were given pink glasses to wear. After continuous use of the glasses for two months, a significant decrease in photophobia was observed in these patients.

## Discussion

In this study, we identified two Pakistani families with autosomal recessive ACHM. All affected individuals had an early onset of nystagmus, photophobia and low visual acuity with color blindness. In these two families, novel mutations were identified in *CNGA3* (c.822G>T, p.R274S) and *CNGB3* (c.1825delG, p.V609WfsX9).

There are six cyclic nucleotide gated (CNG) channels present in mammals, and these are divided in two subfamilies, the A subunits (CNGA1–4) and the B subunits (CNGB1 and CNGB3). The CNGA3 and CNGB3 channels play a critical role in cone-mediated vision, which is required for central and color vision, as well as visual acuity. *CNGA3* and *CNGB3* encode the cone CNG channel subunits [22] that are able to form heterotetramers [23], although CNGA3 subunits may also form homotetramers [24]. Mutations in these two genes have been shown to result in different forms of ACHM and cone dystrophy [23].

In *CNGA3*, the p.R274S mutation is located in a conserved region (Figure 2B), which is part of the fourth transmembrane helix (S4) [17]. The arginine residue at this position is conserved among the CNG alpha subunits of rods (CNGA1) and olfactory neurons (CNGA2), Shaker K^+^ channels and HCN channels [25]. Mutations in the S4 domain have been shown to cause failure in cellular channel processing. Mutant channel proteins are not glycosylated, and do not reach the surface plasma membrane; they remain trapped in the endoplasmic reticulum, and are most likely misfolded [25].

The c.1825delG mutation in exon 16 of *CNGB3* should in theory give rise to nonsense-mediated decay of the mutant RNA, and can, therefore, be considered a loss-of-function mutation. In case there is residual *CNGB3* mRNA, the predicted truncated protein (p.V609WfsX9) lacks the conserved cyclic nucleotide-monophosphate (cNMP) domain. The cNMP domain is the binding site for cyclic nucleotides, and is involved in the cyclic nucleotide mediated activation of the protein. It is hypothesized that both putative effects of this variant will lead to the loss-of-function of CNGB3.

Identification of causative mutations aided in the correct diagnosis of ACHM in these two Pakistani families, which had previously been diagnosed to suffer from retinal dystrophy. After the correct re-diagnosis of the disease, the use of pink glasses reduced the photophobia to some extent in the affected individuals of family RP26. Due to the use of these dark colored pink glasses, the light exposure was reduced, resulting in increased scotopic vision that helped the patients to see objects at a distance, even during the day time. In this study, we report the first genetic screening and use of dark glasses as supportive therapy for ACHM in Pakistan, a disease that is often misdiagnosed.
